# Supplementary material for: MetaQTL: a package of new computational methods for the meta-analysis of QTL mapping experiments
Source: BMC Bioinformatics. 2007 Feb 8;8:49. doi: 10.1186/1471-2105-8-49 (PMC1808479; doi:10.1186/1471-2105-8-49)
Supplement: Additional File 7 — MetaQTL Package : jar file and tutorial. This Zip archive contains both the MetaQTL JAR file and the files of the tutorial. [file 1471-2105-8-49-S7.zip › org.inra.metaqtl/doc/org/metaqtl/graph/GraphFactory.html]

GraphFactory


|  |  |  |
| --- | --- | --- |
| **Overview** \| **Package** \| **Class** \| **Use** \| **Tree** \| **Deprecated** \| **Index** \| **Help** | |  |
| **PREV CLASS**   **NEXT CLASS** | **FRAMES**    **NO FRAMES**     **All Classes** |  |
| SUMMARY: NESTED \| FIELD \| CONSTR \| METHOD | DETAIL: FIELD \| CONSTR \| METHOD |  |


---


## org.metaqtl.graph Class GraphFactory

```
java.lang.Object
  org.metaqtl.graph.GraphFactory
```

---

``` public class GraphFactory extends java.lang.Object ```

---

| **Field Summary** | |
| --- | --- |
| `static int` | `BOX_HEIGHT` |
| `static int` | `BOX_WIDTH` |
| `static int` | `HEIGHT` |
| `static int` | `WIDTH` |


**Constructor Summary**

`GraphFactory()`


| **Method Summary** | |
| --- | --- |
| `static java.awt.image.BufferedImage` | `getImage(ChromGraph[] chroms)` |

**Methods inherited from class java.lang.Object**

`clone, equals, finalize, getClass, hashCode, notify, notifyAll, toString, wait, wait, wait`

**Field Detail**

### WIDTH

```
public static int WIDTH
```

---


### HEIGHT

```
public static int HEIGHT
```

---


### BOX\_WIDTH

```
public static int BOX_WIDTH
```

---


### BOX\_HEIGHT

```
public static int BOX_HEIGHT
```


**Constructor Detail**

### GraphFactory

```
public GraphFactory()
```


**Method Detail**

### getImage

```
public static java.awt.image.BufferedImage getImage(ChromGraph[] chroms)
```


---


|  |  |  |
| --- | --- | --- |
| **Overview** \| **Package** \| **Class** \| **Use** \| **Tree** \| **Deprecated** \| **Index** \| **Help** | |  |
| **PREV CLASS**   **NEXT CLASS** | **FRAMES**    **NO FRAMES**     **All Classes** |  |
| SUMMARY: NESTED \| FIELD \| CONSTR \| METHOD | DETAIL: FIELD \| CONSTR \| METHOD |  |


---
